# Supplementary material for: Homeostatic plasticity and emergence of functional networks in a whole-brain model at criticality
Source: Sci Rep. 2018 Oct 24;8:15682. doi: 10.1038/s41598-018-33923-9 (PMC6200722; doi:10.1038/s41598-018-33923-9)
Supplement: Supplementary file 1 — Supplementary information [file 41598_2018_33923_MOESM1_ESM.pdf]

Homeostatic plasticity and emergence of functional networks in a whole-brain model at criticality

(Supporting Figures)

Rodrigo P. Rocha[1,2,3,*], Loren Koçillari[2,3], Samir Suweis[2,3], Maurizio Corbetta[3,4,5], Amos Maritan[2,3]

[1]Department of Physics, School of Philosophy, Sciences and Letters of Ribeirão Preto, University of São Paulo, Ribeirão Preto, SP, Brazil.

[2]Dipartimento di Fisica e Astronomia, Università di Padova and INFN, via Marzolo 8, I-35131 Padova, Italy.

[3]Padova Neuroscience Center, Università di Padova, Padova, Italy.

[4]Dipartimento di Neuroscienze, Università di Padova, Padova, Italy.

[5]Departments of Neurology, Radiology, Neuroscience, and Bioengineering, Washington University, School of Medicine, St. Louis, USA.

[*] Correspondence to rodrigo.rocha@ufsc.br

August 20, 2018

References

1. A. Ponce-Alvarez, Gustavo Deco, Patric Hagmann, G. Luca Romani, Dante Mantini, Maurizio Corbetta. Resting-State Temporal Synchronization Networks Emerge from Connectivity Topology and Heterogeneity. PLoS Comput Biol 11(2): e1004100 (2015).

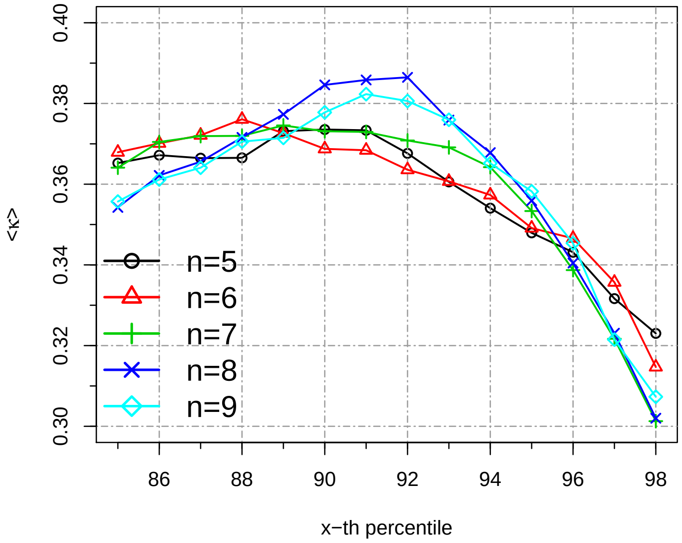

Figure 1: Spatial ICA (sICA) applied to empirical resting state BOLD time-series from the Corbetta et. al. dataset[1]. Overall match $\langle\kappa\rangle$ (Cohen's Kappa) between simulated RSNs and a template of well-established human RSNs as a function of the x-th percentile used to threshold and binarize the simulated RSNs. We considered different values of n, i.e., the number of independent components used to decompose the data, in order to find the dyad of parameters that maximizes $\langle\kappa\rangle$. We found a maximum ($\langle\kappa\rangle \approx 0.39$) at $n = 8$ and the 92-th percentile. We then fixed these values when extracting the RSNs from the simulated dynamics.

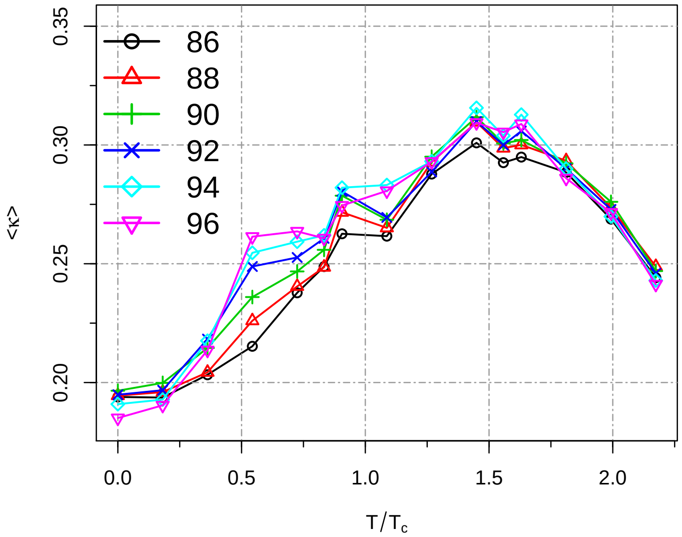

Figure 2: Simulated RSNs using sICA for the not-normalized input matrix W with $n = 8$ components. Overall match $\langle \kappa \rangle$ as a function of T/T_c for different values of the x-th percentile. The highest match is achieved close to the 92-th percentile, in agreement with the previous figure. As discussed in the main text, finite size effects prevent the highest match to coincide with the peak of $\langle S_2 \rangle$.

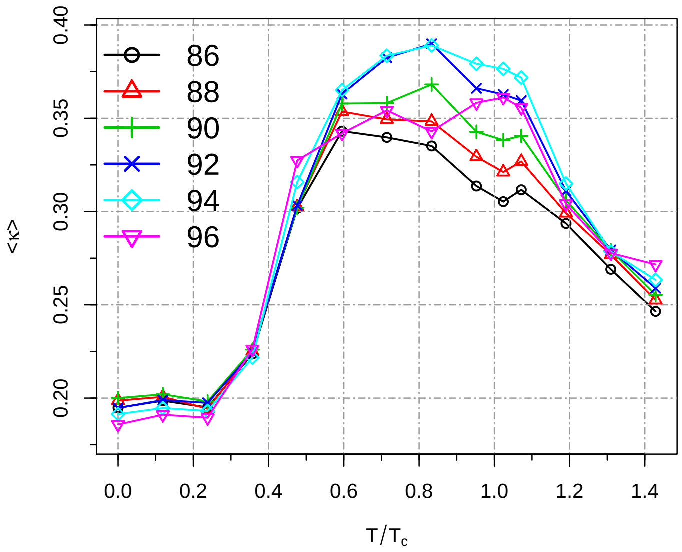

Figure 3: The same as in the previous figure but with the normalized input matrix $\widetilde{W}$. Normalization of the nodes excitatory input causes a substantial improvement of the simulated RSNs maps as compared with the not-normalized input matrix W. The highest match is now more close to the critical point and again it happens in proximity of the 92-th percentile.
